# The Construction and Preclinical Evaluation of Antitumor Activity of a Novel MIgG-OXA ADC in Lung Adenocarcinoma

**DOI:** 10.32604/or.2026.080413

**Published:** 2026-07-16

**Authors:** Haijun Sun, Wenyue Yan, Zhanyu Li, Qintian Li, Qilong Du, Li Xu, Wanwei Cao, Junrong Yang, Xilan Yang, Jun Chen, Yuan Mao, Wen Huang

**Affiliations:** 1 Department of Thoracic Surgery, The First People’s Hospital of Lianyungang, The First Affiliated Hospital of Kangda College of Nanjing Medical University, Lianyungang, China; 2 Department of Geriatric Palliative Care, Thoracoabdominal Oncology Center, The Fourth Affiliated Hospital of Nanjing Medical University, Nanjing, China; 3 Department of Pathology, the Fifth Affiliated Hospital, Sun Yat-sen University, Zhuhai, China; 4 Department of Geriatric Oncology, Thoracoabdominal Oncology Center, The Fourth Affiliated Hospital of Nanjing Medical University, Nanjing, China; 5 Department of Pathology, Jiangsu Cancer Hospital, Affiliated Cancer Hospital of Nanjing Medical University, Nanjing, China; 6 Department of Pathology, The Fourth Affiliated Hospital of Nanjing Medical University, Nanjing, China; 7 Department of Respiratory Medicine, Jiangsu Province Geriatric Hospital, Geriatric Hospital of Nanjing Medical University, Nanjing, China; 8 Department of Hematology and Oncology, Jiangsu Province Geriatric Hospital, Geriatric Hospital of Nanjing Medical University, Nanjing, China

**Keywords:** Melanoma-associated antigen-A1 (MAGE-A1), immunoglobulin G (IgG), oxaliplatin (OXA), antibody-drug conjugates (ADCs)

## Abstract

**Background:** The melanoma-associated antigen-A1 (MAGE-A1) demonstrates tumor-restricted expression patterns in diverse malignancies, positioning it as an attractive therapeutic target. This investigation aimed to engineer and validate a novel antibody-drug conjugate with oxaliplatin targeting MAGE-Al (MIg-OXA), a novel antibody-drug conjugate targeting MAGE-A1, while assessing its therapeutic potential against MAGE-A1-expressing lung adenocarcinoma through both cellular and animal models. **Methods:** We generated a MAGE-A1-specific immunoglobulin G (IgG) antibody (MIgG) and subsequently conjugated it with oxaliplatin (OXA) to produce MIgG-OXA. The conjugate’s binding specificity and cellular uptake were verified through cell-based enzyme-linked immunosorbent assay (ELISA), flow cytometric analysis, and immunofluorescence microscopy. Functional assessments included Cell Counting Kit-8 (CCK-8) viability assays, transwell migration studies, apoptosis detection, and antibody-dependent cell-mediated cytotoxicity (ADCC) evaluation to determine anti-neoplastic activity in cultured cells. Therapeutic efficacy in living organisms was examined using lung adenocarcinoma (LUAD) xenograft-bearing athymic mice. **Results:** Our data confirmed successful synthesis and validation of MIgG-OXA, which demonstrated selective recognition of MAGE-A1-expressing LUAD cell populations and facilitated controlled OXA release. When compared to unconjugated OXA, MIgG-OXA displayed enhanced tumor suppression in both cultured LUAD cells and transplanted tumor models. **Conclusion:** These findings collectively indicate that MIgG-OXA holds substantial promise as a precision therapeutic approach for patients harboring MAGE-A1-positive LUAD.

## 1 Introduction

Lung cancer (LC) remains the predominant cause of cancer-associated deaths throughout China [1,2]. Among non-small cell lung cancer (NSCLC) histological subtypes, lung adenocarcinoma (LUAD) and lung squamous cell carcinoma (LUSC) constitute the two principal categories, with LUAD being particularly characterized by delayed diagnosis, invasive growth patterns, and propensity for early dissemination [3,4]. Recent decades have witnessed transformative advances in LUAD management through enhanced understanding of oncogenic driver alterations and identification of immune checkpoint molecules, culminating in meaningful improvements in clinical outcomes [5,6]. Nevertheless, therapeutic resistance and suboptimal treatment durability persist as formidable obstacles requiring innovative solutions [7]. Consequently, developing novel therapeutic modalities for LUAD patients represents an urgent clinical imperative [8].

Antibody-drug conjugates (ADCs) constitute a rapidly advancing therapeutic class that merges the target specificity of monoclonal antibodies with the cytotoxic potency of chemotherapeutic compounds [9]. Through chemical linker technology, cytotoxic payloads are conjugated to antibodies, permitting selective delivery to antigen-expressing malignant cells while minimizing systemic toxicity and enhancing therapeutic indices [10,11]. ADCs have exhibited remarkable therapeutic potential across various malignancies, particularly solid neoplasms, including breast, gastric, and lung carcinomas [12,13]. Therefore, identifying suitable molecular targets remains a critical determinant for successful ADC development.

The melanoma-associated antigen (MAGE) protein family comprises a large group of cancer-testis antigens, with Type I-MAGE members displaying restricted expression predominantly in malignant tissues, apart from physiological expression in male germline cells and placental structures [14]. Within Type I-MAGE proteins, the MAGE-A subfamily has garnered attention as a significant oncogenic contributor, with mounting evidence supporting its involvement in neoplastic progression and potential as an immunotherapeutic target [15,16]. Notably, MAGE-A1 exhibits tumor-selective expression across numerous solid malignancies [17,18]. In lung cancer pathology, Jungbluth and colleagues detected MAGE-A1 expression in approximately 30% of cases using polymerase chain reaction (PCR)-based methodologies [19], whereas Ayyoub et al. documented prevalence exceeding 40% in NSCLC specimens through immunohistochemical (IHC) evaluation [20]. Building upon these observations, our research team previously engineered a human anti-MAGE-A1 single-chain variable fragment (scFv)-based immunotoxin that exhibited significant anti-neoplastic activity in hepatocellular carcinoma (HCC) cellular models [21].

In the current investigation, we sought to engineer and characterize MIgG-OXA, a novel MAGE-A1-directed ADC derived from our previously developed anti-MAGE-A1 scFv-based antibody. Our specific objectives were to determine whether MIgG-OXA could: (1) selectively recognize MAGE-A1-positive LUAD cells, (2) efficiently deliver oxaliplatin (OXA), and (3) demonstrate enhanced anti-tumor activity in cellular and animal models. Our working hypothesis posited that MIgG-OXA would exhibit superior tumor-suppressive efficacy relative to free OXA in MAGE-A1-expressing LUAD experimental systems.

## 2 Materials and Methods

### 2.1 Bioinformatics Analyses of MAGE-A1 in LC

We initially queried the GeneCards database to investigate the expression pattern of MAGE-A1 (http://www.genecards.org). The Human Protein Atlas (HPA) database was subsequently used to assess MAGE-A1 expression across various LC cell lines (http://www.proteinatlas.org). Afterward, the “Gene” module of the TIMER 2.0 database was employed to analyze the expression of MAGE-A1 in different solid tumors, including LC (http://timer.comp-genomics.org/). Finally, the Gene Expression Profiling Interactive Analysis (GEPIA) database (http://gepia.cancer-pku.cn/) and the Kaplan–Meier plotter database (http://kmplot.com/analysis/) were utilized to evaluate the prognostic significance of MAGE-A1 in LC.

### 2.2 Cell Lines and Reagents

The LUAD cell line H1299 (SCSP-589) and the mouse LUAD cell line LA795 (SCSP-5502) were purchased from the Cell Bank of Shanghai Institute of Biochemistry and Cell Biology (Shanghai, China). HEK 293 Freestyle cells (SCSP-5425.1, Shanghai, China) were cultured at 37°C in FreeStyle™ 293 Expression Medium (12338018, Gibco, San Jose, USA) for antibody expression [22]. All cells have undergone mycoplasma testing and cell line authentication. OXAwas purchased from Shanghai YuanYe Biological Technology Company (B25381, Shanghai, China). Antibody of 4′,6-diamidino-2-phenylindole (DAPI) (ab104139) and cMet-PE (ab310915) were obtained from Abcam (Cambridge, USA). Short-hairpin RNA (shRNA)-mediated knockdown lentiviral plasmids and packaging vectors were prepared as previously described (Supplementary Table S1), to generate the MAGE-A1 knockdown cell line (MAGE-A1-kd. Briefly, shRNA targeting MAGE-A1 (shMAGE) was cloned into pLKD-CMV-G&PR-U6-shRNA (4912, Obio Technology), and the recombinant lentiviral plasmid was subsequently co-transfected. After transfection, the culture supernatants containing lentiviral particles were collected, clarified, and used to infect target cells. Following lentiviral infection, successfully transduced cells were selected with puromycin to establish a stable MAGE-A1-kd cell line [23].

### 2.3 Optimization and Preparation of MAGE-A1-IgG

MAGE-A1-IgG was prepared based on a previously reported MAGE-A1-scFv fragment [21] developed by our research group. The MAGE-A1-scFv amino acid sequence was demonstrated in Supplementary Table S2. To extend antibody half-life, six candidate Fc mutation sets predicted to enhance FcRn binding were evaluated by molecular modeling and docking [24]. Based on the calculated interaction energies, the YTE mutation (M252Y/S254T/T256E) was selected for construction of MAGE-A1-IgG. Protein structure data of FcRn were obtained from the Protein Data Bank (PDB, pdb_00001e4j, https://www.rcsb.org/) and molecular modeling and docking were performed using Discovery Studio 2019 (Dassault Systèmes, BIOVIA Corp., San Diego, USA), as described in our previous study [25]. Following optimization, the variable heavy (VH) and variable light (VL) region sequences derived from the MAGE-A1-scFv were separately amplified by In-Fusion PCR [26], and cloned into the eukaryotic expression vector backbones pFUSE-CHIg-hG1 and pFUSE-CLIg-hκ (InVivogen Grand Island, NY, USA), respectively. Briefly, the pFUSE-CHIg-hG1 and pFUSE-CLIg-hκ vectors were linearized with FspI and BmtI, and the amplified inserts were assembled into the corresponding vectors using an In-Fusion Cloning Kit according to the manufacturer’s instructions (638944, In-Fusion Snap Assembly Master Mix, TAKALA, Mountain View, USA). The affinity of MAGE-A1-IgG for recombinant MAGE-A1 protein (P1285, Fine Biotech, Wuhan, China) was measured using a BLItz instrument (ForteBio, USA). Protein A biosensors were pre-equilibrated in buffer for 10 min and loaded with purified MAGE-A1-IgG at concentrations of 1, 10, 20, 30, and 50 μg/mL. The biosensors were subsequently exposed to recombinant MAGE-A1 protein at 50 μg/mL, and binding kinetics were analyzed using BLItzPro 1.0 software. The equilibrium dissociation constant (K_D_) value was calculated automatically by the software.

### 2.4 Conjugation of MAGE-A1-IgG to OXA (MIgG-OXA)

OXA was first chemically derivatized before antibody conjugation. Briefly, OXA was dissolved in distilled water and reacted with H_2_O_2_ at 70°C for 5 h under light protection, followed by stirring overnight at room temperature. The reaction product was collected by filtration, washed sequentially with ice-cold water, hexanol, and diethyl ether, and dried to obtain a white powder. This intermediate was then reacted with succinic anhydride in dimethyl sulfoxide (DMSO) at 70°C for 24 h under light protection. After removal of the solvent under reduced pressure, the product was precipitated with acetone and collected as a light brown powder. The resulting OXA derivative was dissolved in phosphate buffered saline (PBS) (pH 8.0). OXA was then activated with 1-Ethyl-3-(3-dimethylaminopropyl) carbodiimide (EDC) and N-Hydroxysuccinimide (NHS) to generate an NHS-ester intermediate, which was then added dropwise to MAGE-A1-IgG in phosphate buffered saline (PBS) (pH 8.0). The mixture was incubated for 20 h at 4°C with gentle rotation. The reaction mixture was then purified using a 10-kDa centrifugal ultrafiltration device at 4000× *g* for 20 min at 4°C to remove unreacted OXA and small-molecule byproducts. In the original procedure, the crude conjugate was further dialyzed against PBS (pH 7.4) at 4°C for 24 h with buffer replacement every 4 h to remove residual small-molecule reactants, followed by sterile filtration and concentration under cold conditions. The conjugate was assessed via high-performance liquid chromatography (HPLC, Waters, Milford, MA, USA) using a C18 column (4.6 × 250 mm, 5 μm) with UV detection at 280 nm to evaluate integrity and aggregation. Protein concentration was determined using a micro-bicinchoninic acid protein (BCA) assay kit (PA115-01, TIANGEN, Beijing, China), and OXA concentration was measured by flame atomic absorption spectrometry (FAAS) in accordance with the general rules of GB/T 9723-88 using an atomic absorption spectrometer (Japan) [26].

### 2.5 Immunoprecipitation and Mass Spectrometric Analysis

Protein A/G magnetic beads were pre-washed twice with binding buffer and resuspended before use. MAGE-A1-IgG or MIgG-OXA was diluted in binding buffer to a final concentration of 500 μg/mL and incubated with the beads at room temperature for 15 min on a rotary mixer to allow antibody–bead binding. After magnetic separation and removal of the unbound fraction, antigen-containing samples were added to the bead–antibody complexes and incubated overnight at 4°C with gentle rotation to ensure sufficient antigen binding. The beads were then washed three times with washing buffer. After addition of 25 μL of 1× sodium dodecyl sulfate-polyacrylamide gel electrophoresis (10% SDS-PAGE) loading buffer and heating at 95°C for 10 min, the supernatant was collected following magnetic separation and resolved by SDS-PAGE. Protein bands were excised, destained, reduced, alkylated, and digested with trypsin. The resulting peptides were then subjected to mass spectrometric analysis (Agilent, Santa Clara, USA) as previously described [26].

### 2.6 Effect of pH on Stability of MIgG-OXA

Buffer solutions (0.10 M) covering pH 4.0–10.0 were prepared according to the European Pharmacopoeia (8th edition, EDQM, Strasbourg, 2014) using different buffer systems appropriate for the target pH range. MIgG-OXA was diluted to a final concentration of 5.00 mg/mL and incubated at 37°C with constant stirring at 100 rpm. Samples were collected at 0.5, 1, 2, 4, 8, 12, 24, 48, 72, 96, 120, 144, and 192 h, with replacement of the sampled volume using fresh prewarmed buffer. The elution profile was determined by HPLC, and degradation percentage was calculated as [1 − *A*_t_/*A*_0_] × 100, where *A*_t_ represents the intact conjugate peak area at each time point and *A*_0_ represents that of the control sample at time 0 [27].

### 2.7 ELISA and Internalization Assays

Human MAGE-A1 enzyme-linked immunosorbent assay (ELISA) kit was purchased from MyBioSource (MBS3800683, San Diego, USA). For the ELISA assay, recombinant human MAGE-A1 protein (Fine Biotech) was diluted in coating buffer to 1 μg/mL and added to 96-well plates at 100 μL/well, followed by incubation overnight at 4°C. The plates were washed three times with PBST and blocked with 200 μL/well of 5% skim milk overnight at 4°C. MAGE-A1-IgG, MIgG-OXA, or an isotype control-IgG was then added at the indicated dilutions (1:1000 to 1:64,000) and incubated for 2 h at 37°C. After six washes with PBST, HRP-conjugated goat anti-human Fab diluted 1:4000 in 5% skim milk was added at 50 μL/well and incubated for 1 h at 37°C. Absorbance was measured at 450 nm. For the internalization assay, cells were seeded at 1 × 10^5^ cells/well in 24-well plates and cultured for 24 h. Cells were then incubated with MIgG-OXA (200 μL/well; final concentration 100 μg/mL) and collected at the indicated time points.

For flow cytometric analysis, cells were washed with pre-cooled PBS, blocked with 5% skim milk for 1 h at 37°C, and then incubated with FITC-conjugated goat anti-human secondary antibody (1:50, ab98594, Abcam), for 1 h at 4°C in the dark. After two additional washes with pre-cooled PBS, cells were resuspended in 500 μL flow buffer and analyzed within 1 h on a FACS Calibur flow cytometer (342976, BD Biosciences, San Jose, CA, USA). The internalization percentage was calculated based on mean fluorescence intensities (MFIs). For visualization, cells were first incubated with MIgG-OXA to allow surface binding, then shifted to permit internalization. After washing, cells were labeled with hFc-FITC (A18818, Thermo Fisher Scientific, Waltham, USA) and counterstained with 4′,6-diamidino-2-phenylindole (DAPI). Following PBS-Tween (PBST) washes, images were acquired using a fluorescence microscope (BX53, Olympus, Tokyo, Japan).

### 2.8 Tumor-Inhibitory Effectiveness of MIgG-OXA In Vitro

Cell proliferation, migration, and apoptosis were examined by counting kit-8 (CCK-8), transwell and flow cytometry (FCM) analyses, to evaluate the tumor-inhibitory characteristics of MIgG-OXA in LUAD cell lines, as detailed protocol was described in a previous study [28].

For the CCK-8 assay (CK04-500T, Dojindo, Kumamoto, Japan), cells were seeded into 96-well plates at 1 × 10^4^ cells/well and incubated for 24 h, followed by treatment with MIgG-OXA, MAGE-A1-IgG, or OXA at the indicated concentrations for 48 h. Thereafter, 10 μL CCK-8 reagent and 100 μL serum-free medium (KBM 581, Corning, New York, USA) were added to each well, and the plates were incubated for 2 h at 37°C before absorbance was measured at 450 nm (800TS, Biotek, Vermont, USA).

For the transwell assay, cells were resuspended in serum-free medium and seeded into the upper chamber (200 μL), while 600 μL complete medium containing 20% fetal bovine serum (A5669701, Gibco, New York, USA) was added to the lower chamber. After 24 h of incubation, non-migrated cells on the upper surface were removed, and the migrated cells were fixed with methanol for 10 min, stained with 0.1% crystal violet for 30 min, and counted in six randomly selected microscopic fields (IX51, Olympus, Tokyo, Japan).

For apoptosis analysis, cells were seeded into 6-well plates at 5 × 10^5^ cells/well, treated with MIgG-OXA, MAGE-A1-IgG, or OXA for 24 h, and then harvested together with floating cells. The cells were washed twice with cold PBS, resuspended in binding buffer at 1 × 10^6^ cells/mL, stained with Annexin V and propidium iodide for 15 min at room temperature in the dark, and analyzed by flow cytometry. Antibody-dependent cell-mediated cytotoxicity (ADCC) was evaluated using a lactate dehydrogenase (LDH) release assay. H1299 cells were used as target cells. MIgG-OXA or MAGE-A1-IgG was added to the target cells at indicated concentrations ranging from 10^−2.5^ to 10^0^ μM. Control IgG and OXA were included as controls. Peripheral blood mononuclear cells (PBMCs) were then collected and added to achieve effector to target (E: T) ratios of 10:1, 20:1, and 40:1. After co-incubation, LDH release in the culture supernatant was measured using the LDH Cytotoxicity Assay Kit (C0018M, Beyotime, Shanghai, China) according to the manufacturer’s protocols. Cytotoxicity was calculated using the following formula: Cytotoxicity(%) = 100 × [(experimental release − effector spontaneous release − target spontaneous release)/(target maximum release − target spontaneous release)] [26].

### 2.9 Tumor-Inhibitory Effectiveness of MIgG-OXA In Vivo

Male 3-week-old BALB/c nude mice with a body weight of ~20 g were purchased from SLAC Laboratory Animal (Shanghai, China) were used to establish LUAD xenografts, and kept under specific pathogen-free conditions [29].

A total of 48 mice were included in the study. LA795 cells (5 × 10^6^ in 0.1 mL) were subcutaneously injected into the right flank of each mouse. When tumors reached approximately 100 mm^3^, the mice were randomly assigned to eight groups (n = 6 per group): OXA (1 mg/kg), OXA (2.5 mg/kg), OXA (5 mg/kg), OXA (10 mg/kg), MIgG-OXA (equivalent to OXA 1 mg/kg), MIgG-OXA (equivalent to OXA 2.5 mg/kg), MIgG-OXA (equivalent to OXA 5 mg/kg), and vehicle 0.9% saline solution. Outcome assessors were blinded to group allocation during tumor volume measurements and histological evaluations. Control IgG (self-made Trop2-IgG, 10 mg/kg) was prepared and administered using the same route and schedule as MIgG-OXA. All treatments were administered via intravenous injection. Tumor length and width were measured using a Vernier caliper every 3 days, and tumor volume was calculated using the formula: tumor volume (mm^3^) = L × W^2^/2, where L represents the longest tumor diameter, and W represents the shortest diameter. Body weight and general condition were monitored throughout the experiment. Predefined humane endpoints were established prior to study initiation and included any of the following criteria: tumor volume exceeding 2000 mm^3^, body weight loss greater than 20% of baseline, signs of severe distress (labored breathing, inability to ambulate, or persistent recumbency), or skin ulceration overlying the tumor. Mice meeting any of these criteria were immediately euthanized by CO_2_ inhalation followed by cervical dislocation, in accordance with institutional guidelines. At the end of the study, mice were euthanized, and tumors were excised for further evaluation. For the bioluminescence imaging (BLI) study, LA795-LUC cells were used to establish LUAD xenografts using the same inoculation procedure. After inoculation, tumor-bearing mice were divided randomly into 4 treatment groups (OXA (5 mg/kg), MIgG-OXA (equivalent to OXA 1 mg/kg), MAGE-A1-IgG (10 mg/kg), and Control IgG (self-made Trop2-IgG, 10 mg/kg)). Bioluminescence signals were acquired and analyzed using a Xenogen IVIS 200 Imaging System (Caliper Life Sciences, Hopkinton, MA, USA). All animal experiments were conducted in accordance with the Public Health Service Policy and were approved by the Animal Care and Use Committee of The Fourth Affiliated Hospital of Nanjing Medical University (SFY20220907-K150).

### 2.10 Statistical Analysis

Quantitative data are presented as mean ± standard error of the mean (SEM), and analyzed by Variance Analysis and SNK-q test. MAGE-A1expression levels in LUAD cell lines were estimated by the two-tailed unpaired Student’s *t*-test. Differences in therapeutic effects *in vitro* and *in vivo* were analyzed by two-way analysis of variance (ANOVA). A *p*-value < 0.05 was considered statistically significant. All statistical analyses were performed using SPSS version 18.0 (SPSS Inc., Armonk, NY, USA) and STATA version 17.0 (Stata Corporation, College Station, USA).

## 3 Results

### 3.1 Characteristics of MAGE-A1 Expression by Bioinformatics Analyses

**Figure 1:**
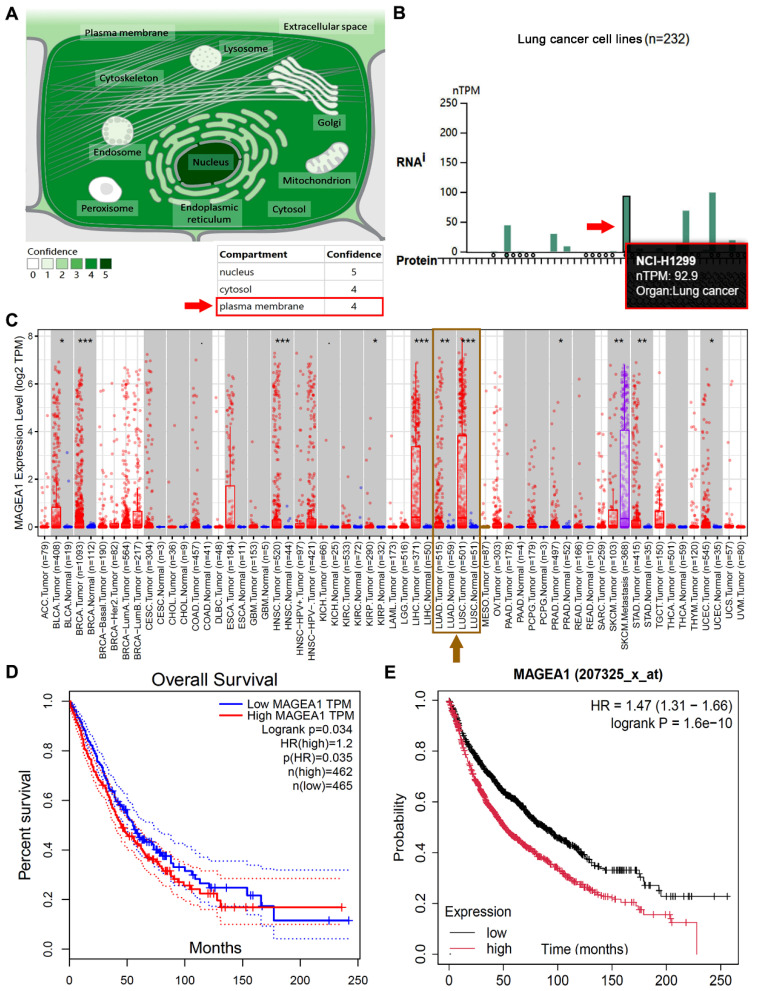
Bioinformatics analyses of MAGE-A1. (**A**) The GeneCard database showed the major expression modes of MAGE-A1 in cells. One predominant subcellular localization of MAGE-A1 was in the plasma membranes (confidence 4, marked by a red frame). (**B**) The Human Protein Atlas (HPA) demonstrated the expression pattern of MAGE-A1 across various lung cancer (LC) cell lines and confirmed that the H1299 cell line is positive for MAGE-A1 (marked by a red frame). (**C**) The TIMER2.0 database illustrated that the RNA expression of MAGE-A1 in lung cancer (LUAD: lung adenocarcinoma; LUSC: lung squamous cell carcinoma) tissues was significantly higher than in the corresponding noncancerous tissues (marked by a brown frame). *p < 0.05; **p < 0.01; ***p < 0.001. (**D**) Survival data from the GEPIA database (*p* = 0.034) confirmed that high MAGE-A1 expression was associated with unfavorable overall survival in LC. (**E**) Survival data from the K/Mplotter database (*p* = 1.6 × 10^−10^) validated that high MAGE-A1 expression was associated with unfavorable overall survival in LC.

The GeneCards database was queried to investigate the expression pattern of MAGE-A1. The results indicated that MAGE-A1 is expressed on the membrane of cancer cells (expression confidence = 4), a key feature for designing an antibody-based ADC (Fig. 1A). Analysis of the HPA database revealed positive MAGE-A1 expression in the LUAD H1299 cell line (Fig. 1B). TIMER2.0 database data showed that MAGE-A1 RNA expression in lung cancer tissues (LUAD and LUSC) was significantly higher than in corresponding noncancerous tissues (Fig. 1C). Survival analyses from the GEPIA (Fig. 1D) and KMplot (Fig. 1E) databases confirmed that high MAGE-A1 expression was associated with poorer overall survival in LC (*p* = 0.034 and *p* = 1.6 × 10^−10^, respectively).

### 3.2 Optimization and Construction of MAGE-A1-IgG

**Figure 2:**
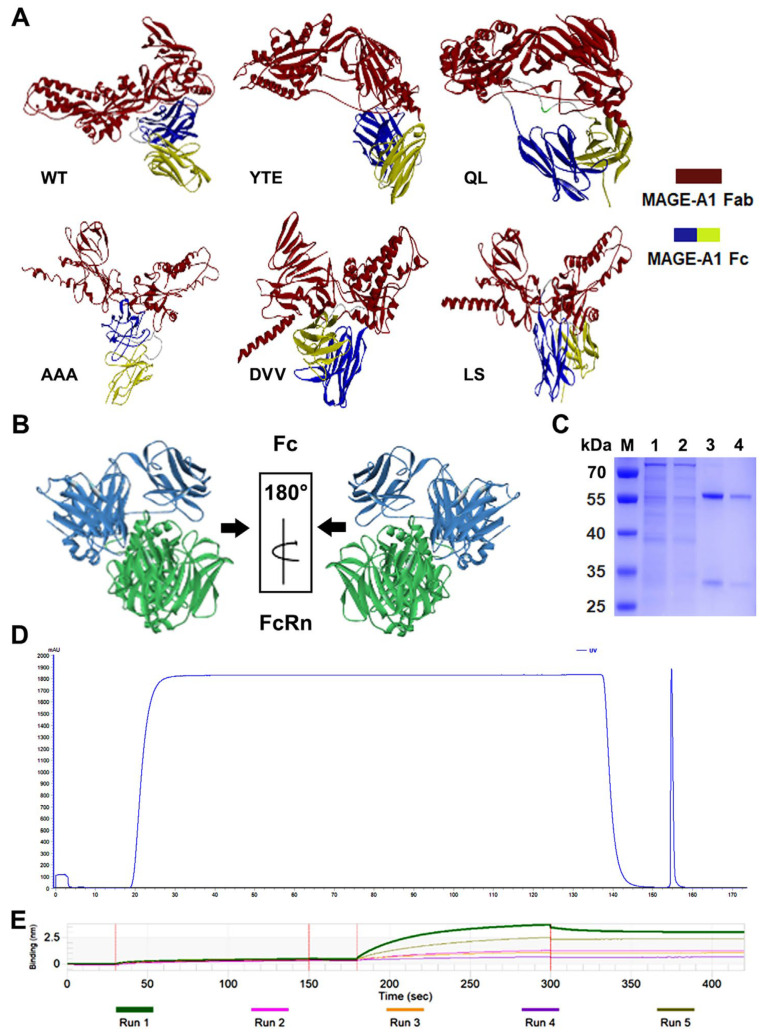
Optimization and preparation of MAGE-A1-IgG. (**A**) Six sets of Fc residues were designed to mutate to alter FcRn interactions and improve IgG characteristics (WT, YTE, QL, AAA and DVV). (**B**) The 3D diagram showed the binding mode of the Fc–FcRn interaction with the YTE mutation. (**C**) SDS-PAGE confirmed that MAGE-A1-IgG was successfully purified. M: Marker Fermentas SM0671; lane 1: 293F Cell supernatant (transfected with MAGE-A1-IgG vectors); lane 2: flow through; lane 3: purified MAGE-A1-IgG antibody eluate; lane 4: purified isotype control antibody eluate. (**D**) UV curve of MAGE-A1-IgG purification. (**E**) The equilibrium dissociation constant (K_D_) for MAGE-A1-IgG was 9.58 × 10^−9^ M.

Six sets of Fc residues were identified that, when mutated, were predicted to modulate FcRn interactions and enhance IgG properties (Fig. 2A). These sets included residues directly involved in FcRn binding, as well as peripheral and buried sites capable of reshaping the interaction interface and influencing the dynamics of the relevant helical segment. Among the six sets, the YTE mutation was selected for the optimization and construction of MAGE-A1-IgG due to its optimal interaction energy (Table 1). Fig. 2B illustrates the binding mode of the Fc–FcRn interaction with the YTE mutation. Fig. 2C,D confirm that MAGE-A1-IgG was successfully purified and validated. A BLItz affinity assay determined the equilibrium dissociation constant (K_D_) of MAGE-A1-IgG to be 9.58 × 10^−9^ M (Fig. 2E).

**Table 1:** Interaction energy comparison of different Fc mutations for MAGE-A1-IgG.

MAGE-A1-IgG Modification	ID	Interaction Energy (kcal/mol)
Wild Type	MAGE-A1-IgG (WT)	E = −2023.52517 kcal/mol
M252Y/S254T/T256E	MAGE-A1-IgG (YTE)	E = −2630.75783 kcal/mol
T132Q/M219L	MAGE-A1-IgG (QL)	E = −2476.24784 kcal/mol
T53A/E83A/N343A	MAGE-A1-IgG (AAA)	E = −2218.27696 kcal/mol
H258D/T298V/A324V	MAGE-A1-IgG (DVV)	E = −1913.34395 kcal/mol
M338L/N354S	MAGE-A1-IgG (LS)	E = −2362.60554 kcal/mol

### 3.3 Preparation and Verification of MIgG-OXA

**Figure 3:**
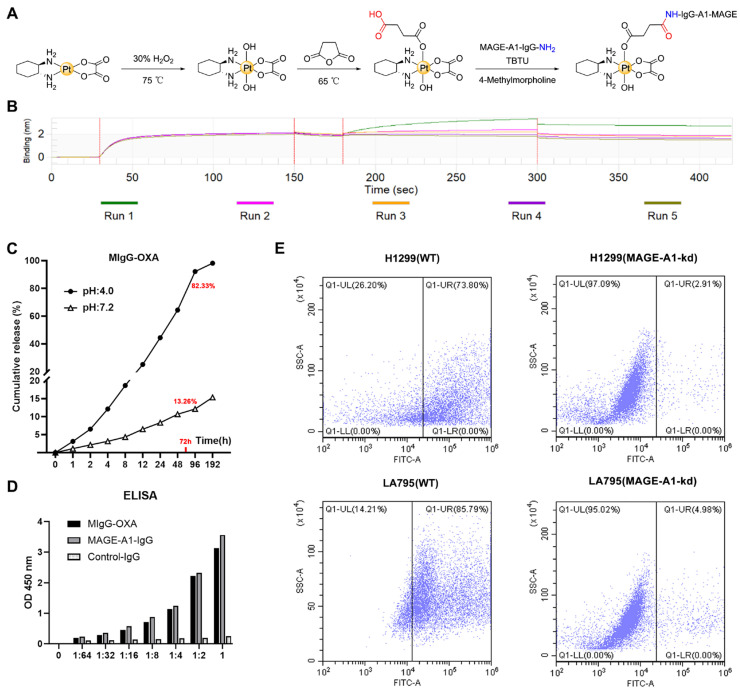
Conjugation and characterization of MIgG-OXA. (**A**) Using EDC/NHS-mediated carbodiimide chemistry, OXA was covalently linked to MAGE-A1–IgG via lysine ε-amines to yield MIgG-OXA. (**B**) The K_D_ of MIgG-OXA was calculated by a BLITz affinity assay, and the value was 7.42 × 10^−9^ M. (**C**) In the pH stability assay, MIgG-OXA remained largely stable at neutral pH (7.2), showing only 15.4% cumulative release at 192 h. By contrast, under acidic conditions (pH 4.0) the conjugate dissociated rapidly, releasing MIgG-OXA into the medium and reaching 82.33% cumulative release at 72 h, far exceeding the 13.26% release at 72 h in neutral buffer. (**D**) ELISA assays established the specific affinity of MIgG-OXA for MAGE-A1, following a dose-dependent trend. (**E**) Flow cytometry provided additional evidence that MIgG-OXA specifically targets LUAD cells expressing MAGE-A1.

MIgG-OXA was prepared by conjugating MAGE-A1-IgG to OXA via the ε-amino groups of lysine using EDC/NHS chemistry and analyzed by Reverse Phase High Performance Liquid Chromatography (RP-HPLC). The synthetic schemes of MAGE-A1-IgG with OXA was shown in Fig. 3A. The concentration of MAGE-A1-IgG was measured using a BCA assay, while OXA concentration was determined by FASS. The final average drug-to-antibody ratio (DAR) of MIgG-OXA was 2.32 (Table 2). A BLItz affinity assay determined the K_D_ of MIgG-OXA to be 7.42 × 10^−9^ M (Fig. 3B). In the pH stability assay, MIgG-OXA demonstrated good stability under neutral conditions (pH 7.2), with a cumulative release of 15.4% at 192 h (Fig. 3C). In contrast, under acidic conditions (pH 4.0), MIgG-OXA rapidly dissociated from the conjugation site, reaching a cumulative release of 82.33% at 72 h, which was markedly higher than the 13.26% release observed under neutral conditions over the same period. Fig. 3D from the ELISA assay demonstrates that MIgG-OXA specifically binds to the MAGE-A1 protein in a dose-dependent manner. Flow cytometry analysis (Fig. 3E) further confirmed that MIgG-OXA selectively recognizes MAGE-A1-positive LUAD cells. In H1299 wild-type (WT) and H1299 MAGE-A1-kd cells treated with MIgG-OXA, the percentages of positive cells in H1299 WT cells was significantly higher than in H1299 MAGE-A1-kd cells (73.80% vs. 2.91%, *p* < 0.05). These results were corroborated in LA795 WT and LA795 MAGE-A1-kd cells, showing MFI values of 85.79% versus 4.98%, respectively (*p* < 0.05).

**Table 2:** OXA/MAGE-A1-IgG ratio of MIgG-OXA.

Concentration	Value
MAGE-A1-IgG concentration (mg/mL)*	3.21
OXA concentration (μg/mL)**	19.73
OXA/MAGE-A1-IgG ratio	2.32

*MAGE-A1-IgG concentration was detected by BCA Protein Assay Kit. **OXA concentration was determined by FAAS.

### 3.4 Internalization of MIgG-OXA in LUAD Cell Lines

**Figure 4:**
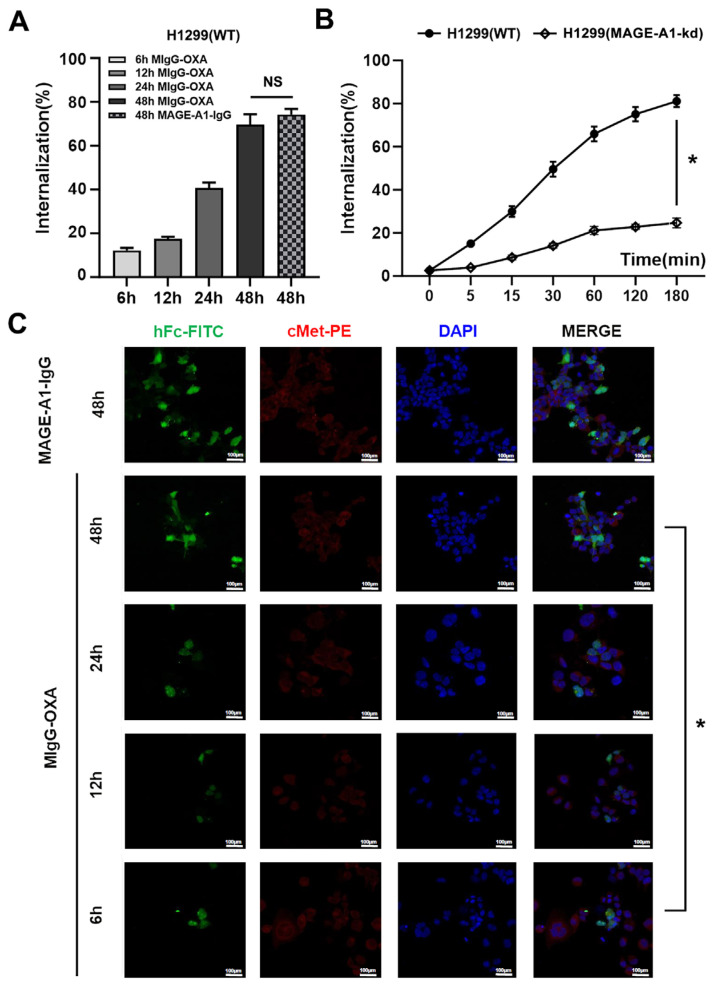
Internalization test of MIgG-OXA. (**A**) Internalization test demonstrated that MIgG-OXA undergoes significant, time-dependent uptake into H1299 (WT) cells over 48 h, reaching approximately 70% by that point. NS: no significance. (**B**) Binding value of internalization test was substantially diminished in H1299 (MAGE-A1-kd) compared with H1299 (WT) cells (24.6 ± 2.25% vs. 81.1 ± 2.74%). *p < 0.05. (**C**) Immunofluorescence analysis corroborated the rapid and efficient internalization of MIgG-OXA into MAGE-A1-positive LUAD cells with a time-dependent increase after incubation. *p < 0.05.

To assess whether MIgG-OXA is internalized after binding to cell-surface MAGE-A1, uptake was quantified by flow cytometry and confirmed by fluorescence microscopy. Flow cytometric analysis (Fig. 4A) revealed significant, time-dependent internalization of MIgG-OXA into H1299 cells (WT, MAGE-A1 positive) over 48 h, with uptake reaching approximately 70% at this time point. Fig. 4B shows that MIgG-OXA binding to H1299 MAGE-A1-kd cells was markedly reduced compared with H1299 WT cells (24.6 ± 2.25% vs. 81.1 ± 2.74%). Consistently, fluorescence microscopy demonstrated a time-dependent increase in intracellular fluorescence in H1299 WT cells following MIgG-OXA incubation (Fig. 4C). Together, these results indicate that MIgG-OXA is rapidly and efficiently internalized into MAGE-A1-positive LUAD cells.

### 3.5 MIgG-OXA Shows Anti-Tumor Activity Against LUAD Cell In Vitro

**Figure 5:**
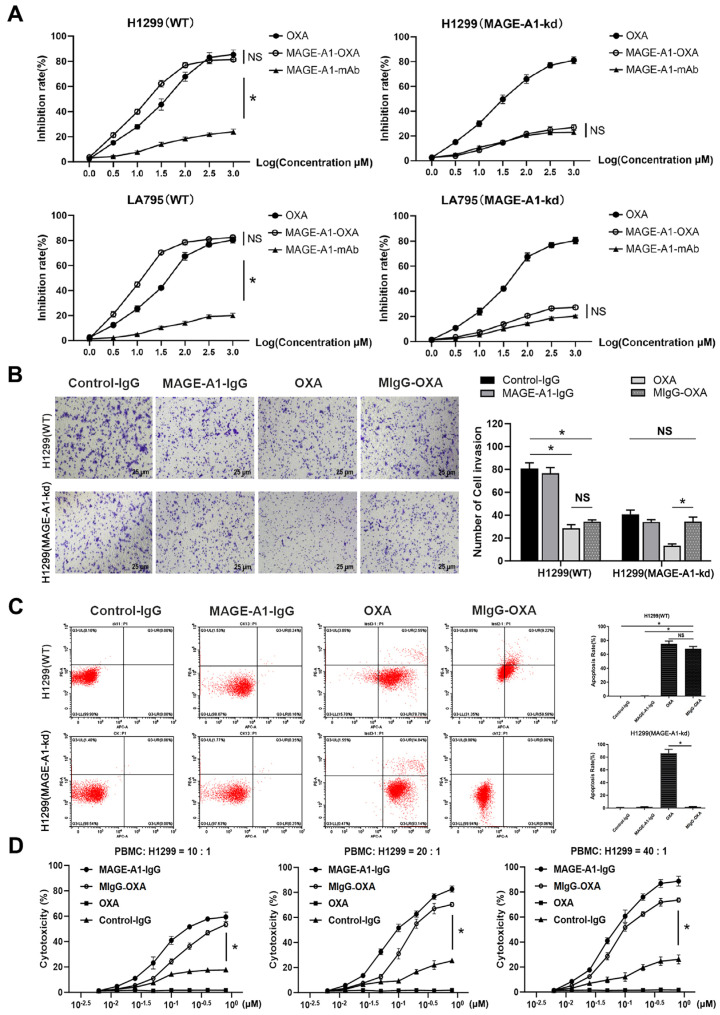
Tumor inhibitory effectiveness of MIgG-OXA *in vitro*. (**A**) When normalized to OXA equivalents, MIgG-OXA achieved lower IC50s than OXA in MAGE-A1-positive WT cells 12.28 μM vs. 52.43 μM for H1299 and 9.36 μM vs. 48.35 μM for LA795. In H1299 (MAGE-A1-kd) and LA795 (MAGE-A1-kd) cell lines, MIgG-OXA activity was greatly reduced and showed no difference with that of OXA. (**B**) Both MIgG-OXA and OXA significantly curtailed invasion in H1299 (WT) cells. By contrast, MIgG-OXA rarely influenced on invasion in H1299 (MAGE-A1-kd) cells when compared to OXA. (**C**) Flow cytometry documented a substantial increase in apoptosis following OXA or MIgG-OXA treatment, while MIgG-OXA did not induce apoptosis in the MAGE-A1-deficient cell lines relative to OXA. (**D**) MIgG-OXA and MAGE-A1-IgG produced dose-dependent ADCC in H1299 (WT) with similar efficacy. In comparison, OXA scarcely induced ADCC. *p < 0.05, NS: no significance.

*In vitro*, the cytotoxicity of MIgG-OXA was assessed using a CCK-8 assay in H1299 (WT and MAGE-A1-kd) and LA795 (WT and MAGE-A1-kd) cell lines. The calculated half-maximal inhibitory concentrations (IC_50_) are summarized in Table 3. As shown in Fig. 5A, OXA exhibited clear cytotoxicity in both cell lines. On an OXA-equivalent basis, MIgG-OXA displayed significantly enhanced cytotoxicity against MAGE-A1-positive H1299 WT cells, with IC50 values of 12.28 μM for MIgG-OXA compared with 52.43 μM for OXA. Similarly, in LA795 WT cells, the IC50 values were 9.36 μM for MIgG-OXA and 48.35 μM for OXA, consistent with the previously determined DAR of 2.32. In contrast, MIgG-OXA exhibited minimal cytotoxicity against MAGE-A1 knockdown cells (IC_50_ > 1000 μM). These results indicate that MIgG-OXA cytotoxicity is specific to MAGE-A1-positive LUAD cells and is significantly reduced in cells with low or absent MAGE-A1 expression.

**Table 3:** The impact of various drugs on the proliferation of H1299 and LA795 cell lines.

Cell Lines	IC50 (μM)		
	OXA	MAGE-A1-IgG	MIgG-OXA
H1299 (WT), mean ± SEM	52.43 ± 1.04	>1000	12.28 ± 1.02
H1299 (MAGE-A1kd), mean ± SEM	42.72 ± 1.02	>1000	>1000
LA795 (WT), mean ± SEM	48.35 ± 1.06	>1000	9.36 ± 1.03
LA795 (MAGE-A1-kd), mean ± SEM	35.47 ± 1.07	>1000	>1000

### 3.6 Influence of MIgG-OXA on Migration, Apoptosis and ADCC In Vitro

In the transwell assay, both OXA and MIgG-OXA significantly reduced the invasion of H1299 WT cells (*p* < 0.05; Fig. 5B). In contrast, MIgG-OXA had minimal effect on the invasion of H1299 MAGE-A1-kd-cells compared with OXA (*p* < 0.05), indicating that MIgG-OXA specifically inhibits migration in MAGE-A1-positive LUAD cells. In the apoptosis assay, flow cytometry showed a marked increase in apoptotic rates following treatment with OXA or MIgG-OXA (Fig. 5C). However, in H1299 MAGE-A1-kd cells, MIgG-OXA failed to induce apoptosis relative to OXA. For ADCC analysis, both MIgG-OXA and MAGE-A1-IgG triggered dose-dependent ADCC against H1299 WT cells with comparable efficacy, whereas OXA induced negligible ADCC in all groups (Fig. 5D).

### 3.7 MIgG-OXA Inhibited Xenograft Development In Vivo

**Figure 6:**
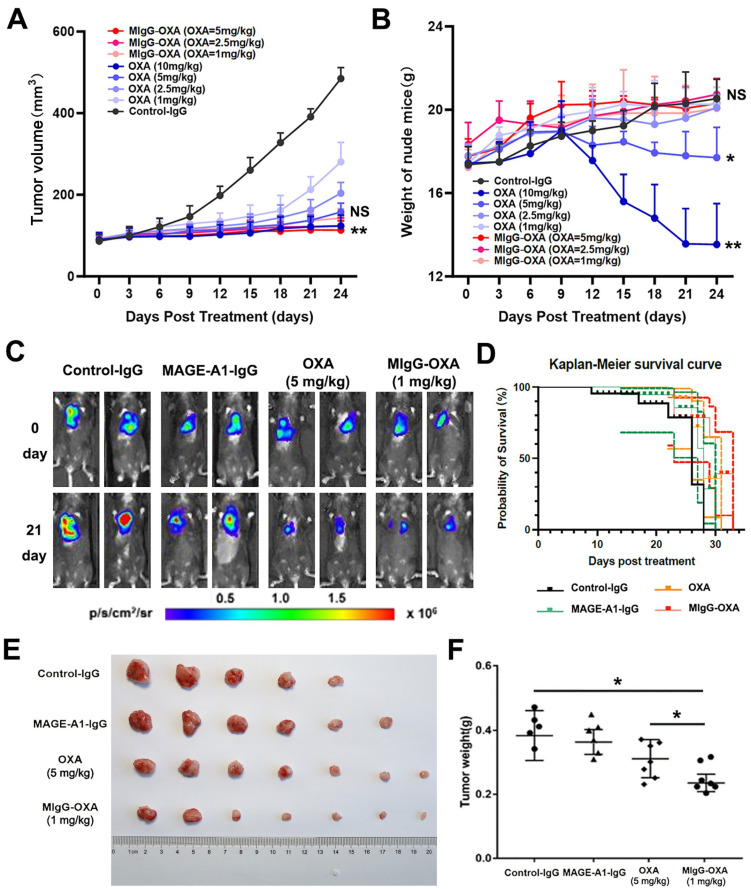
Tumor inhibitory effectiveness of MIgG-OXA *in vivo*. (**A**) Relative to Control-IgG, every MIgG-OXA dose and OXA at ≥ 2.5 mg/kg produced significant tumor suppression. The strongest effect was observed with MIgG-OXA at 5 mg/kg (OXA-equivalent), and MIgG-OXA at 1 mg/kg (OXA-equivalent) achieved tumor control comparable to OXA at 5 mg/kg. (**B**) Body weights were tracked longitudinally, revealing a significant reduction in the high-dose OXA (5 and 10 mg/kg) group compared with Control-IgG. In contrast, weights in mice receiving MIgG-OXA at 1 mg/kg remained statistically indistinguishable from those of Control-IgG. (**C**) In LA795-LUC xenograft model, MIgG-OXA or OXA treatment yielded markedly weaker and smaller bioluminescent signals versus Control-IgG and MAGE-A1-IgG. (**D**) MIgG-OXA significantly extended survival status compared with the other groups. (**E**) Comparison of xenograft tumor morphology after mice sacrifice. The size of xenograft tumors was significantly suspended after MIgG-OXA administration (**F**) Tumor weights showed that MIgG-OXA selectively targeted and robustly inhibited the growth of MAGE-A1-positive xenografts *in vivo*. *p < 0.05, **p < 0.01, NS, no significance.

To evaluate tumor-inhibitory effectiveness, *in vivo* experiments were performed using four OXA dose groups (1, 2.5, 5, and 10 mg/kg) and three MIgG-OXA dose groups (1, 2.5, and 5 mg/kg OXA-equivalent). Compared with the control IgG, all MIgG-OXA doses and OXA at doses above 2.5 mg/kg showed significant antitumor activity. Notably, MIgG-OXA at 5 mg/kg OXA-equivalent produced the most pronounced tumor suppression, while MIgG-OXA at 1 mg/kg OXA-equivalent exhibited tumor-inhibitory efficacy comparable to OXA at 5 mg/kg (Fig. 6A). Fig. 6B further shows that mice treated with high-dose OXA (5 and 10 mg/kg) experienced significant weight loss due to anorexia induced by the treatment. The LA795-LUC cell line was subsequently used to establish subcutaneous xenografts. MIgG-OXA (1 mg/kg) and OXA (5 mg/kg) were selected to repeat the *in vivo* experiments using bioluminescence imaging. Fig. 6C shows that tumor bioluminescence signals were markedly weaker and occupied smaller volumes following treatment with MIgG-OXA or OXA compared with the control IgG and MAGE-A1-IgG groups. Additionally, MIgG-OXA significantly prolonged the survival of mice bearing LA795-LUC tumors relative to the other three treatment groups (Fig. 6D). Analysis of tumor morphology (Fig. 6E) and weight (Fig. 6F) further confirmed that MIgG-OXA specifically targeted and effectively inhibited the growth of MAGE-A1-positive xenograft tumors *in vivo*.

## 4 Discussion

Tumor cells exhibit high adaptability and often develop resistance to initial therapeutic regimens, requiring higher doses to achieve effective eradication [30]. However, many currently available cytotoxic drugs lack selectivity and have a narrow therapeutic window. For example, platinum-based chemotherapeutic regimens are widely used as first-line chemotherapy for LUAD [31], yet their clinical utility is constrained by drug resistance development and adverse effects, including acute nephrotoxicity, severe hepatorenal reactions, and chronic neurotoxicity [32]. Recently, the development of antibody–drug conjugates (ADCs) has garnered substantial attention. For instance, T-DXd has dramatically improved outcomes in HER2-positive metastatic breast cancer [33]. In NSCLC, ADCs have demonstrated reduced toxicity and enhanced tumor-targeting efficacy compared with conventional chemotherapeutics in multiple preclinical and clinical studies [34,35].

Given that MAGE-A1 has been characterized as a potential target in LC [36], we performed a comprehensive series of bioinformatics analyses to substantiate its candidacy as a biomarker in LUAD. These data robustly indicate that MAGE-A1 represents a promising therapeutic target for LUAD treatment. Additionally, the fragment crystallizable (Fc) region of antibodies can engage Fc-gamma Receptors (FcγRs) expressed on innate effector cells, extending antibody functionality beyond binding or neutralization to encompass recruitment of phagocytes and NK cells, as well as complement activation [37]. Building upon the previously prepared MAGE-A1-scFv fragment, we employed biomedical tools to optimize the IgG structure via Fc-FcRn interactions to enhance its performance. Among six modification candidates, the YTE mutation was selected for the optimization and construction of MAGE-A1-IgG. These findings align with previous reports showing that the YTE variant extends antibody half-life, confirming the feasibility of modulating antibody pharmacokinetics [38].

We subsequently designed and prepared the novel MIgG-OXA, consisting of MAGE-A1-IgG conjugated to OXA via an amino–carboxyl chemical-bond linker. In ADCs, the linker plays important roles in maintaining stability, minimizing nonspecific release prior to internalization, and enabling efficient payload delivery [39]. HPLC analysis confirmed the successful synthesis of MIgG-OXA, and FASS, along with BCA protein assays indicated a DAR of 2.32, within an acceptable range [40]. In OXA release experiment, MIgG-OXA remained stable under neutral conditions, avoiding degradation during transport in the bloodstream when intravenously administered into the systemic circulation under neutral conditions. Conversely, OXA was rapidly released from the MIgG-OXA linker within the tumor microenvironment, exerting its cytotoxic effects.

Moreover, ELISA, flow cytometry, and immunofluorescence analyses confirmed that MIgG-OXA retained high binding affinity for the MAGE-A1 protein, indicating that conjugation did not alter the antigen-recognition properties of the MAGE-A1-IgG antibody. Collectively, these data demonstrate that MIgG-OXA can specifically recognize, bind to, and be efficiently internalized by MAGE-A1-positive LUAD cells. Consistently, Shi et al. developed conjugates using a MAGE-A1 peptide and a TLR2 agonist, which effectively promoted dendritic cell maturation, CD8^+^ T-cell activation, and tumor growth inhibition [41]. These findings are in line with the results of the present study and strongly support the rationale and potential of MAGE-based cancer immunotherapy.

*In vitro*, MIgG-OXA exhibited markedly greater cytotoxicity toward MAGE-A1-positive H1299 (WT) and LA795 (WT) cells compared with their MAGE-A1-kd counterparts. For example, in LA795 WT cells, MIgG-OXA achieved tumor-inhibitory efficacy at a lower dose (IC_50_ = 9.36 μM) comparable to that of OXA alone (IC_50_ = 48.35 μM). Phenotypic assays further demonstrated that MIgG-OXA significantly inhibited cell migration, promoted apoptosis, and induced ADCC. Similarly, a recent study reported that a Trop2-based ADC (Dato-DXd) markedly enhanced apoptosis and elicited robust ADCC in Trop2-expressing tumor models [42].

*In vivo*, although OXA alone inhibited tumor growth in xenograft models, its efficacy was lower than that of MIgG-OXA. MIgG-OXA achieved the highest tumor inhibition rate in LUAD xenograft mice, accompanied by the longest survival time and better general condition. In comparison, high-dose OXA treatment caused apparent reduction in body weight in mice. These findings suggest that MIgG-OXA administration alleviates the systemic toxicity associated with OXA. Collectively, the results highlight the advantages of MIgG-OXA, including potent efficacy, low toxicity, and high tumor specificity. Similarly, our previous research has demonstrated the the therapeutic potential of a Trop2-based ADC for pancreatic cancer treatment [43]. Yu et al. also reported that a novel anti-CD56 antibody-MMAE conjugate exhibited strong anti-cancer effects both *in vivo* and *in vitro* with excellent tolerability [44].

Several limitations in the present study should be acknowledged. First, we did not include additional human or murine LUAD cell lines, which would have strengthened the robustness of our conclusions. Second, OXA was selected for the preparation of MIgG-OXA rather than cisplatin or carboplatin mainly because OXA offers distinct immunologic advantages, including the robust induction of immunogenic cell death and activation of the cGAS–STING pathway in tumors, which is particularly beneficial in the context of cancer immunotherapy. Several studies have successfully employed OXA as an antitumor agent in LC research [45,46]. Third, a full pharmacokinetic (PK) study, toxicity analysis and the mechanism of MIgG-OXA activity in LUAD were not investigated, as the primary objective of this present study was the successful construction of MIgG-OXA and a preliminary evaluation of its tumor-inhibitory effects. Future studies with more detailed PK validation, toxicity evaluation and mechanistic investigation are warranted to confirm and extend these current findings.

## 5 Conclusions

In conclusion, we successfully constructed a novel ADC, MIgG-OXA, which retained high binding affinity for MAGE-A1 and demonstrated superior anti-tumor efficacy compared with free OXA both *in vitro* and *in vivo*. These findings suggest that MIgG-OXA represents a promising targeted therapeutic strategy for MAGE-A1-expressing LUAD patients and warrants further clinical investigation.

## Data Availability

The datasets generated and/or analyzed during this current study are available from the corresponding author on reasonable request.

## Supplementary Materials

The supplementary material is available online at https://www.techscience.com/doi/10.32604/or.2026.080413/s1.

## Abbreviation

ADC	antibody–drug conjugate
ADCC	antibody-dependent cell-mediated cytotoxicity
BCA	bicinchoninic acid (BCA) protein assay
CCK-8	Cell Counting Kit-8
DAR	drug-to-antibody ratio
DAPI	4′,6-diamidino-2-phenylindole
EGFR	epidermal growth factor receptor
ELISA	enzyme-linked immunosorbent assay
FBS	fetal bovine serum
FCM	flow cytometry
GEPIA	Gene Expression Profiling Interactive Analysis
HPA	The Human Protein Atlas
IC50	half maximal inhibitory concentration
IHC	immunohistochemistry
kd	knockdown
LC	lung cancer
LDH	lactate dehydrogenase
LUAD	lung adenocarcinoma
LUSC	lung squamous cell carcinoma
MAGE-A1	melanoma-associated antigen-A1
NSCLC	non-small cell lung cancer
OXA	oxaliplatin
PBS	phosphate-buffered saline
PBST	PBS with Tween-20
RPMI	RPMI-1640 medium
SDS-PAGE	sodium dodecyl sulfate–polyacrylamide gel electrophoresis
SEM	standard error of the mean
shRNA	short hairpin RNA
WT	wild-type

## References

[1] Hendriks LEL , Remon J , Faivre-Finn C , Garassino MC , Heymach JV , Kerr KM , et al. Non-small-cell lung cancer. Nat Rev Dis Primers. 2024; 10: 71. doi:10.1038/s41572-024-00551-9. 39327441

[2] Zou J , Lu Y , Li J , Zhou Z , Peng F , Qiu P , et al. Progression on mechanism and therapeutic implications of neddylation in lung cancer. Oncol Res. 2026; 34( 2): 9. doi:10.32604/or.2025.071940. PMC12848757 41613808

[3] Normanno N , Morabito A , Rachiglio AM , Sforza V , Landi L , Bria E , et al. Circulating tumour DNA in early stage and locally advanced NSCLC: Ready for clinical implementation? Nat Rev Clin Oncol. 2025; 22( 3): 215– 31. doi:10.1038/s41571-024-00985-w. 39833354

[4] Li X , Han M , Zhang L , Xie X , Li C , Zhang H , et al. Gymconopin C exhibits anti-non-small cell lung cancer effect by regulating miR-6777-5p/ADRB2 pathway to promote mitophagy. J Adv Res. 2025. Online ahead of print. doi:10.1016/j.jare.2025.12.023. 41423048

[5] Wood C , Lyniv L , Isaacs JM , Kaufman JM , Oduah EI , Clarke J , et al. Perioperative pembrolizumab in early-stage non-small cell lung cancer (NSCLC): Safety, efficacy, and exploratory biomarker analysis. J Immunother Cancer. 2025; 13( 2): e010395. doi:10.1136/jitc-2024-010395. 39904561 PMC11795402

[6] Sun H , Li M , Huang W , Zhang J , Wei S , Yang Y , et al. Thoracic radiotherapy improves the survival in patients with EGFR-mutated oligo-organ metastatic non-small cell lung cancer treated with epidermal growth factor receptor-tyrosine kinase inhibitors: A multicenter, randomized, controlled, phase III trial. J Clin Oncol. 2025; 43( 4): 412– 21. doi:10.1200/JCO.23.02075. 39374473

[7] Bai Y , Liu X , Zheng L , Wang S , Zhang J , Xiong S , et al. Comprehensive profiling of EGFR mutation subtypes reveals genomic-clinical associations in non-small-cell lung cancer patients on first-generation EGFR inhibitors. Neoplasia. 2023; 38: 100888. doi:10.1016/j.neo.2023.100888. 36804751 PMC9975296

[8] Xie J , Xu J , Tian Z , Liang J , Tang H . Extended insights into advancing multi-omics and prognostic methods for cancer prognosis forecasting. Front Biosci. 2025; 30( 8): 44091. doi:10.31083/FBL44091. 40917070

[9] Mountzios G , Saw SPL , Hendriks L , Menis J , Cascone T , Arrieta O , et al. Antibody-drug conjugates in NSCLC with actionable genomic alterations: Optimizing smart delivery of chemotherapy to the target. Cancer Treat Rev. 2025; 134: 102902. doi:10.1016/j.ctrv.2025.102902. 39978083

[10] Dumontet C , Reichert JM , Senter PD , Lambert JM , Beck A . Antibody–drug conjugates come of age in oncology. Nat Rev Drug Discov. 2023; 22( 8): 641– 61. doi:10.1038/s41573-023-00709-2. 37308581

[11] Colombo R , Tarantino P , Rich JR , LoRusso PM , & de Vries EGE . The journey of antibody-drug conjugates: Lessons learned from 40 years of development. Cancer Discov. 2024; 14( 11): 2089– 108. doi:10.1158/2159-8290.CD-24-0708. 39439290

[12] Drago JZ , Modi S , Chandarlapaty S . Unlocking the potential of antibody-drug conjugates for cancer therapy. Nat Rev Clin Oncol. 2021; 18( 6): 327– 44. doi:10.1038/s41571-021-00470-8. 33558752 PMC8287784

[13] Yin Q , Zhang Y , Xie X , Hou M , Chen X , Ding J . Navigating the future of gastric cancer treatment: A review on the impact of antibody-drug conjugates. Cell Death Discov. 2025; 11( 1): 144. doi:10.1038/s41420-025-02429-5. 40188055 PMC11972320

[14] Weon JL , Potts PR . The MAGE protein family and cancer. Curr Opin Cell Biol. 2015; 37: 1– 8. doi:10.1016/j.ceb.2015.08.002. 26342994 PMC4688208

[15] Alsalloum A , Shevchenko JA , Sennikov S . The melanoma-associated antigen family a (MAGE-a): A promising target for cancer immunotherapy? Cancers. 2023; 15( 6): 1779. doi:10.3390/cancers15061779. 36980665 PMC10046478

[16] Simister PC , Border EC , Vieira JF , Pumphrey NJ . Structural insights into engineering a T-cell receptor targeting MAGE-A10 with higher affinity and specificity for cancer immunotherapy. J Immunother Cancer. 2022; 10( 7): e004600. doi:10.1136/jitc-2022-004600. 35851311 PMC9295655

[17] Srdelić S , Kuzmić-Prusac I , Spagnoli GC , Juretić A , Čapkun V . MAGE-A4 and MAGE-A1 immunohistochemical expression in high-grade endometrial cancer. Int J Gynecol Pathol. 2019; 38( 1): 59– 65. doi:10.1097/PGP.0000000000000470. 29140883

[18] Chen A , Qiu Y , Yen YT , Wang C , Wang X , Li C , et al. Expression of cancer-testis antigens MAGE-A1, MAGE-A4, NY-ESO-1 and PRAME in bone and soft tissue sarcomas: The experience from a single center in China. Cancer Med. 2025; 14( 7): e70750. doi:10.1002/cam4.70750. 40152485 PMC11951172

[19] Jungbluth AA , Stockert E , Chen YT , Kolb D , Iversen K , Coplan K , et al. Monoclonal antibody MA454 reveals a heterogeneous expression pattern of MAGE-1 antigen in formalin-fixed paraffin embedded lung tumours. Br J Cancer. 2000; 83( 4): 493– 7. doi:10.1054/bjoc.2000.1291. 10945497 PMC2374655

[20] Ayyoub M , Memeo L , Alvarez-Fernández E , Colarossi C , Costanzo R , Aiello E , et al. Assessment of MAGE-a expression in resected non-small cell lung cancer in relation to clinicopathologic features and mutational status of EGFR and KRAS. Cancer Immunol Res. 2014; 2( 10): 943– 8. doi:10.1158/2326-6066.CIR-13-0211. 24866168

[21] Lin H , Mao Y , Zhang DW , Li H , Qiu JR , Zhu J , et al. Selection and characterization of human anti-MAGE-A1 scFv and immunotoxin. Anticancer Agents Med Chem. 2013; 13( 8): 1259– 66. doi:10.2174/18715206113139990134. 23343082

[22] Wang Z , Tang Q , Liu B , Zhang W , Chen Y , Ji N , et al. A SARS-CoV-2 neutralizing antibody discovery by single cell sequencing and molecular modeling. J Biomed Res. 2022; 37( 3): 166– 78. doi:10.7555/JBR.36.20220221. 36992606 PMC10226085

[23] Mao Y , Fan W , Hu H , Zhang L , Michel J , Wu Y , et al. MAGE-A1 in lung adenocarcinoma as a promising target of chimeric antigen receptor T cells. J Hematol Oncol. 2019; 12( 1): 106. doi:10.1186/s13045-019-0793-7. 31640756 PMC6805483

[24] Ghetie V , Popov S , Borvak J , Radu C , Matesoi D , Medesan C , et al. Increasing the serum persistence of an IgG fragment by random mutagenesis. Nat Biotechnol. 1997; 15( 7): 637– 40. doi:10.1038/nbt0797-637. 9219265

[25] Mao Y , Chen Y , Yang X , He Y , Cui D , Huang W , et al. Construction and characterization of a novel secreted MsC-CAR-T cell in solid tumors. Cancer Lett. 2025; 611: 217382. doi:10.1016/j.canlet.2024.217382. 39642980

[26] Ma Y , Zhang M , Wang J , Huang X , Kuai X , Zhu X , et al. High-affinity human anti-c-met IgG conjugated to oxaliplatin as targeted chemotherapy for hepatocellular carcinoma. Front Oncol. 2019; 9: 717. doi:10.3389/fonc.2019.00717. 31428584 PMC6688309

[27] Mohamed HE , Mohamed AA , Al-Ghobashy MA , Fathalla FA , Abbas SS . Stability assessment of antibody-drug conjugate Trastuzumab emtansine in comparison to parent monoclonal antibody using orthogonal testing protocol. J Pharm Biomed Anal. 2018; 150: 268– 77. doi:10.1016/j.jpba.2017.12.022. 29258046

[28] Xiao X , Peng Y , Wang Z , Zhang L , Yang T , Sun Y , et al. A novel immune checkpoint siglec-15 antibody inhibits LUAD by modulating mφ polarization in TME. Pharmacol Res. 2022; 181: 106269. doi:10.1016/j.phrs.2022.106269. 35605813

[29] Lin H , Zhang H , Wang J , Lu M , Zheng F , Wang C , et al. A novel human Fab antibody for Trop2 inhibits breast cancer growth *in vitro* and *in vivo*. Int J Cancer. 2014; 134( 5): 1239– 49. doi:10.1002/ijc.28451. 23982827

[30] Chisholm RH , Lorenzi T , Clairambault J . Cell population heterogeneity and evolution towards drug resistance in cancer: Biological and mathematical assessment, theoretical treatment optimisation. Biochim Biophys Acta. 2016; 1860( 11 Pt B): 2627– 45. doi:10.1016/j.bbagen.2016.06.009. 27339473

[31] Liang J , Bi N , Wu S , Chen M , Lv C , Zhao L , et al. Etoposide and cisplatin versus paclitaxel and carboplatin with concurrent thoracic radiotherapy in unresectable stage III non-small cell lung cancer: A multicenter randomized phase III trial. Ann Oncol. 2017; 28( 4): 777– 83. doi:10.1093/annonc/mdx009. 28137739

[32] Amable L . Cisplatin resistance and opportunities for precision medicine. Pharmacol Res. 2016; 106: 27– 36. doi:10.1016/j.phrs.2016.01.001. 26804248

[33] André F , Hee Park Y , Kim SB , Takano T , Im SA , Borges G , et al. Trastuzumab deruxtecan versus treatment of physician’s choice in patients with HER2-positive metastatic breast cancer (DESTINY-Breast02): A randomised, open-label, multicentre, phase 3 trial. Lancet. 2023; 401( 10390): 1773– 85. doi:10.1016/S0140-6736(23)00725-0. 37086745

[34] Ramlau R , Kowalski DM , Szczęsna A , Szczylik C , Ou Y , Köbel M , et al. Safety of unconventional antibody-drug conjugate L-DOS47 in a phase I/II monotherapy study targeting advanced NSCLC. Front Oncol. 2025; 15: 1544967. doi:10.3389/fonc.2025.1544967. 40860822 PMC12375440

[35] Jin X , Zhao W , Li B , Gu Y , Li Z , Guo W , et al. Efficacy and safety of antibody-drug conjugates in the treatment of non-small cell lung cancer: A systematic review and meta-analysis of prospective clinical trials. Transl Cancer Res. 2025; 14( 9): 5255– 70. doi:10.21037/tcr-2025-376. 41158249 PMC12554454

[36] Hu K , Gao L , Zhang R , Lu M , Zhou D , Xie S , et al. Clinical application of serum seven tumour-associated autoantibodies in patients with pulmonary nodules. Heliyon. 2024; 10( 9): e30576. doi:10.1016/j.heliyon.2024.e30576. 38765082 PMC11098830

[37] Noble A , Paudyal B , Schwartz JC , Mwangi W , Munir D , Tchilian E , et al. Distinct effector functions mediated by Fc regions of bovine IgG subclasses and their interaction with Fc gamma receptors. Front Immunol. 2023; 14: 1286903. doi:10.3389/fimmu.2023.1286903. 38077405 PMC10702552

[38] Booth BJ , Ramakrishnan B , Narayan K , Wollacott AM , Babcock GJ , Shriver Z , et al. Extending human IgG half-life using structure-guided design. mAbs. 2018; 10( 7): 1098– 110. doi:10.1080/19420862.2018.1490119. 29947573 PMC6204840

[39] Tsuchikama K , An Z . Antibody-drug conjugates: Recent advances in conjugation and linker chemistries. Protein Cell. 2018; 9( 1): 33– 46. doi:10.1007/s13238-016-0323-0. 27743348 PMC5777969

[40] Schumacher D , Hackenberger CPR , Leonhardt H , Helma J . Current status: Site-specific antibody drug conjugates. J Clin Immunol. 2016; 36( Suppl 1): 100– 7. doi:10.1007/s10875-016-0265-6. 27003914 PMC4891387

[41] Shi W , Tong Z , Chen S , Qiu Q , Zhou J , Qian H . Development of novel self-assembled vaccines based on tumour-specific antigenic peptide and TLR2 agonist for effective breast cancer immunotherapy via activating CD8^+^ T cells and enhancing their function. Immunology. 2023; 169( 4): 454– 66. doi:10.1111/imm.13643. 36946150

[42] Ettorre VM , Demirkiran C , Bellone S , Hartwich TMP , Greenman M , McNamara B , et al. Effective preclinical activity of datopotamab deruxtecan (Dato-DXd), an ADC targeting trophoblast cell-surface antigen 2 (TROP2), against primary cervical carcinoma cell lines and xenografts. Gynecol Oncol. 2025; 201: 195– 202. doi:10.1016/j.ygyno.2025.08.027. 40907200

[43] Mao Y , Wang X , Zheng F , Wang C , Tang Q , Tang X , et al. The tumor-inhibitory effectiveness of a novel anti-Trop2 Fab conjugate in pancreatic cancer. Oncotarget. 2016; 7( 17): 24810– 23. doi:10.18632/oncotarget.8529. 27050150 PMC5029744

[44] Yu L , Yao Y , Wang Y , Zhou S , Lai Q , Lu Y , et al. Preparation and anti-cancer evaluation of promiximab-MMAE, an anti-CD56 antibody drug conjugate, in small cell lung cancer cell line xenograft models. J Drug Target. 2018; 26( 10): 905– 12. doi:10.1080/1061186X.2018.1450413. 29630426

[45] Bag A , Schultz A , Bhimani S , Stringfield O , Dominguez W , Mo Q , et al. Coupling the immunomodulatory properties of the HDAC6 inhibitor ACY241 with Oxaliplatin promotes robust anti-tumor response in non-small cell lung cancer. Oncoimmunology. 2022; 11( 1): 2042065. doi:10.1080/2162402X.2022.2042065. 35223194 PMC8865306

[46] Ni Y , Li R , Shen X , Yi D , Ren Y , Wang F , et al. Diaphorobacter nitroreducens synergize with oxaliplatin to reduce tumor burden in mice with lung adenocarcinoma. mSystems. 2024; 9( 4): e01323– 23. doi:10.1128/msystems.01323-23. 38483163 PMC11019951

