# Supplementary material for: The Construction and Preclinical Evaluation of Antitumor Activity of a Novel MIgG-OXA ADC in Lung Adenocarcinoma
Source: Oncol Res. 2026 Jul 16;34(8):21. doi: 10.32604/or.2026.080413 (PMC13397353; doi:10.32604/or.2026.080413)
Supplement: Supplementary file 1 [file OncolRes-34-80413-s001.zip › TSP_OR_80413-s001.docx]

Supplementary Table S1. Primer and siRNA sequences

| Primer types | Primer sequences |
| --- | --- |
| MAGE-A1 forward | 5’- CAG CAT TTC TGC CTT TGT -3’ |
| MAGE-A1 reverse | 5’- GCC TTT CCC ACT ACC ATC -3’ |
| MAGE-A1 siRNA | 5’- CCT CGC TGA AAC CAG CTA T -3’ |
| MAGE-A1 siRNA-NC | 5’- TTC TCC GAA CGT GTC ACG T -3’ |

Supplementary Table S2. MAGE-A1-scFv amino acid sequence

| MAGE-A1-scFv amino acid sequence |
| --- |
| >MAGE-A1 VH  EVQLLESGGGLVQPGGSLRLSCAASGFTFSSYAMSWVRQAPGKGLEWVSAISGSGGSTYYADPVKGRFTISRDNSKNTLYLQMNSLRAEDTAVYYCAKLIHDFDYWGQGTLVTVSS |
| >MAGE-A1 VK  DIQMTQSPSSLSASVGDRVTITCRASQSISSYLNWYQQKPGKAPKLLIYRASALQSGVPSRFSGSGSGTDFTLTISSLQPEDFATYYCQQSRKTPHTFGQGTKVEIK |
